# Lateral Access Surgery for the Thoracolumbar Spine

**DOI:** 10.1155/2013/241705

**Published:** 2013-03-06

**Authors:** Luiz Pimenta, William Smith, William Taylor, Juan Uribe

**Affiliations:** ^1^Department of Minimally Invasive Surgery, Instituto de Patologia da Coluna, 041011 000 Sao Paulo, SP, Brazil; ^2^Division of Neurosurgery, UC San Diego, San Diego, CA 92093, USA; ^3^Department of Neurosurgery, University Medical Center and, NNI Research Foundation, Las Vegas, NV 89109, USA; ^4^Department of Neurosurgery and Brain Repair, University of South Florida, Tampa, FL 33612, USA

The advent of minimally invasive surgery has provided surgeons and patients the same good treatment alternatives. Within the field of spine surgery, techniques for lumbar interbody arthrodesis have shown enormous evolution. The retroperitoneal transpsoas lateral approach for the lumbar spine has revolutionized how interbody fusions can be performed more safely and with significantly less morbidity. In recent years, the role of minimally invasive transpsoas interbody fusion is strongly evolving. Lateral retroperitoneal approaches under direct visualization using expandable retractors have gained space and have been utilized for different purposes rather than interbody fusion. The objective of this special issue was to, for the first time in the literature, gather together articles on Lateral Access Surgery (LAS) and assemble unique peer-reviewed literature with basic and advanced reports focused in this emerging area.

The number of scientific articles about minimally invasive spine surgery (MISS) and specially about LAS is growing. The nature of the research tends to go to the same direction and evolve from single-center case reports to a higher level of evidence trials, once a recognized weakness in the MIS literature is the absence of randomized controlled trials. But by the other hand, randomizing patients were found to be challenging faced to patients' expectative and ethical principles. Given the challenges of conducting a randomized controlled trial and although more substantial research remains to be performed, nonrandomized concurrently controlled trials or prospective registries will provide valuable evidence-based medicine data. The level of evidence provided from the clinical and basic studies included in this special issue is not at the highest level of clinical trials, but though they represent different aims and indications for the lateral access, it can identify two patterns across them—safety and efficacy.

 Among the table of content of this issue, V. S. Patel et al. go through overall indications and outcomes, while M. Malham et al. from Australia show results from their initial experience with the technique. The use of an allograft as bone graft option is well reported by A. G. Tohmeh et al. Basic science developments are illustrated with studies on biomechanics of interbody constructions and on the validation of an animal model. First developed for anterior based discectomy and interbody grafting in simple degenerative diseases, LAS has recently gained space for more complex indications. Over the years, it has been possible to address varied and complex scenarios. In this mindset, this special issue brings important reports that discuss advanced surgical applications of LAS—release of the ALL and sagittal reconstruction, degenerative scoliosis, application for revision surgery and thoracolumbar infection, and for L4-5 grade 2 spondylolisthesis.

We look forward to witness the evolution of spine surgery with the evidence based medicine to achieve the highest efficacy and safe to the patient who suffers from any spine-related condition.

*Luiz Pimenta* *Luiz Pimenta* *William Smith* *William Smith* *William Taylor* *William Taylor* *Juan Uribe* *Juan Uribe*

